# Shuangxinfang Prevents S100A9-Induced Macrophage/Microglial Inflammation to Improve Cardiac Function and Depression-Like Behavior in Rats After Acute Myocardial Infarction

**DOI:** 10.3389/fphar.2022.832590

**Published:** 2022-06-24

**Authors:** Yize Sun, Zheyi Wang, Jiqiu Hou, Jinyu Shi, Zhuoran Tang, Chao Wang, Haibin Zhao

**Affiliations:** ^1^ Third Affiliated Hospital, Beijing University of Chinese Medicine, Beijing, China; ^2^ Qilu Hospital, Cheeloo College of Medicine, Shandong University, Jinan, China; ^3^ Oriental Hospital, Beijing University of Chinese Medicine, Beijing, China

**Keywords:** Shuangxinfang, traditional Chinese medicine, acute myocardial infarction, epressive disorder, S100A9, inflammation, microglia, macrophages

## Abstract

**Background:** Depression is a common complication of cardiovascular disease, which deteriorates cardiac function. Shuangxinfang (psycho-cardiology formula, PCF) was reported to alleviate myocardial ischemia injury and improve depression-like behavior. Interestingly, our previous proteomics study predicted that the protein S100A9 appeared as an important target, and macrophage/microglial inflammation might be involved in the process of PCF improving depression induced by acute myocardial infarction (AMI). This study aims to validate the proteomics results.

**Methods:** AMI rat models were established *in vivo*, followed by the administration of PCF or ABR-215757 (also named paquinimod, inhibiting S100A9 binding to TLR4) for 5 days. Forced swimming test (FST) and open field test (OFT) were applied to record depression-like behavior, and echocardiography was employed to evaluate cardiac function. Morphological changes of cardiomyocytes were assessed by HE staining and TUNEL staining on day 7 after cardiac surgery, as well as Masson trichrome staining on day 21. Hippocampal neurogenesis was determined by Nissl staining, while 5-hydroxytryptamine (5-HT), tryptophan/kynurenine ratio, and brain-derived neurotrophic factor (BDNF) in the hippocampus were analyzed as biochemical indicators of depression. We employed RT-qPCR, western blotting, and immunofluorescence to detect the expression of pathway-related genes and proteins. Myocardial and hippocampal expression of inflammatory factors were performed by ELISA. The activation of macrophage and microglia was assessed *via* immunoreaction using CD68 and Iba1, respectively. For *in vitro* confirmation, BV2 cells were primed with recombinant protein S100A9 and then treated with PCF serum or ferulic acid to determine alterations in microglial inflammation.

**Results:** Rats in the AMI group showed heart function deterioration and depression-like behavior. Coronary ligation not only brought about myocardial inflammation, cell apoptosis, and fibrosis but also reduced the neurogenesis, elevated the tryptophan/kynurenine ratio, and decreased the content of 5-HT. PCF could ameliorate the pathological and phenotypic changes in the heart and brain and inhibit the expression of the S100A9 protein, the activation of the microglial cell, and the secretion of IL-1β and TNF-α raised by AMI. ABR-215757 showed therapeutic effect and molecular biological mechanisms similar to PCF. Treatment with PCF serum or ferulic acid *in vitro* was proved to efficiently block the hyperactivation of BV2 cells and increment of cytokine contents induced by recombinant protein S100A9.

**Conclusion:** We identify S100A9 as a novel and potent regulator of inflammation in both the heart and brain. Macrophage/microglia inflammation mediated by S100A9 is considered a pivotal pathogenic in depression after AMI and a major pathway for the treatment of PCF, suggesting that PCF is a promising therapeutic candidate for psycho-cardiology disease.

## 1 Background

The reported prevalence of depression after acute myocardial infarction (AMI) for the last few years varied across studies and generally ranged from 18% to 40% (Smolderen et al., 2017; Feng et al., 2019; Trajanovska et al., 2019; Worcester et al., 2019). The TRIUMPH study, an observational multicenter cohort study published in *Circulation*, which enrolled 4,062 patients with AMI and recognized depression between 24 and 72 h of admission, declared that one-fifth of patients with AMI had significant depressive symptoms (Smolderen et al., 2017). The research assessed depression in patient survivors during hospitalization at 3 and 12 months after AMI, and the three groups presented almost equal representation of depression according to beck depression inventory (BDI) with 34.1%, 30.8%, and 30%, respectively (Trajanovska et al., 2019). These results implied that acute coronary events might directly induce depression, regardless of other socioeconomic factors. It is reported that only patients with incident post-AMI depression, rather than ongoing or recurrent depressions, had an impaired cardiovascular prognosis (de Jonge et al., 2006), suggesting that the pathological mechanism of AMI-induced depression may be different from other types and worthy of further investigation.

Depression has been classified as a risk factor for poor prognosis among patients with cardiovascular diseases, which is closely related to decreased heart rate variability, sympathetic nervous excitement, and ventricular arrhythmias, ulteriorly leading to fatal and non-fatal cardiovascular events, loss of life quality, an increase in healthcare expenditure, and suicide risk (Gehi et al., 2005; Rodrigues et al., 2015; Hawkins et al., 2016; AbuRuz and Al-Dweik, 2018; Wilkowska et al., 2019; Bangalore et al., 2020). Selective serotonin reuptake inhibitors (SSRIs) are currently preferred choices for depressed patients with cardiovascular disease. However, associations of antidepressant treatment with long-term cardiac outcomes in depression following AMI have been inconclusive (Coupland et al., 2016; Kim et al., 2018; Iasella et al., 2019; Kim et al., 2019). It means that new therapeutic strategies still need to be developed to make up for the deficiency of current antidepressants.

Shuangxinfang (psycho-cardiology formula, PCF) consists of four kinds of botanical drugs, including *Salvia miltiorrhiza* Bunge (Lamiaceae; *Salviae miltiorrhizae* radix et rhizoma), the roots and rhizomes of *Chuanxiong Rhizoma* (Umbelliferae; *Ligusticum chuanxiong* Hort.), the bulb of *Lilium pumilum* DC (Liliaceae; Lilii Bulbus), and the dried seeds of *Ziziphi Spinosae Semen* [Rhamnaceae; *Ziziphus jujuba* Mill. var. spinosa (Bunge) Hu ex H.F.Chou], which are beneficial in promoting blood circulation, removing stasis, lifting the spirit, and gaining the vitality to be away from gloomy mood and somatic distress. The main active substances of *Salvia miltiorrhiza* Bunge include the phenolic acids, the diterpenoid tanshinones, and related quinone derivatives (Pang et al., 2016). *Lilium pumilum* DC contains various chemical components, in which steroidal saponins, flavonoids, and polysaccharides are the main active ingredients (Zhou et al., 2021). *Ziziphi Spinosae Semen* contains flavonoids, saponins, alkaloids, and fatty oils (Hua et al., 2021). Besides, 174 components have been identified from *Chuanxiong Rhizoma*, among which phthalides and alkaloids would be the main bioactive ingredients for the pharmacological properties (Chen et al., 2018). Our previous clinical trials have already confirmed that PCF could relieve angina pectoris and improve depressive symptoms (Wang et al., 2021). The pharmacological mechanism of PCF concentrates on the regulation of inflammatory response and the neuroendocrinology system. PCF could inhibit the expression of inflammatory factors such as tumor necrosis factor-α (TNF-α) in AMI rats and meanwhile appease the neural system by modulating the γ-aminobutyric acid (GABA) system (Wang et al., 2019). The above data highlighted a critical role for the PCF in inhibiting inflammation caused by injured myocardium and alleviating depression following AMI.

To systematically identify possible targets and explore the biological mechanism of PCF in depression after AMI, we have performed pharmacoproteomic profiling of the myocardium and hippocampus in rats from the sham, AMI, and PCF groups using label-free liquid chromatography-mass spectrometry (LC-MS/MS) (Sun et al., 2021). The intersection of differentially expressed proteins (DEPs) in the peri-infarct border zone and hippocampus produces a unique protein, that is S100A9, which has become a topic molecule in the cardiovascular field during these years (Nagareddy et al., 2020; Wang et al., 2021). The role of S100A9 in driving inflammatory response after MI has attracted much attention and has been identified as a potential therapeutic target (Li et al., 2019). Also, the alarmin S100A9 mediating neuroinflammation in depressive-like behaviors begins to come into focus (Gong et al., 2018). According to alteration of the proteomics profile in biological fraction and pertinent pathways, macrophage/microglia inflammation might be a biological mechanism for PCF to protect against the pathological progress of depression after AMI. As reported, S100A9 modulates macrophage inflammation in AMI and regulates microglial inflammation in depression (Ma et al., 2017; Marinković et al., 2019), yet the evidence of it is not quite adequate in post-AMI depression. Indeed, the regulation of S100A9 in macrophage/microglia inflammation guides a direction for molecular mechanisms in psycho-cardiology diseases. In this study, systematic experiments were performed in AMI rats with depression-like behavior to verify the hypothesis derived from proteomics.

## 2 Materials and Methods

### 2.1 Preparation of PCF

One dosage of PCF was composed of the roots and rhizomes of *Salvia miltiorrhiza* Bunge (Lamiaceae; *Salviae miltiorrhizae* radix et rhizoma) (Dan Shen, 20 g), the roots and rhizomes of *Chuanxiong Rhizoma* (Umbelliferae; *Ligusticum chuanxiong* Hort.) (Chuan Xiong, 12 g), the bulb of *Lilium pumilum* DC (Liliaceae; Lilii Bulbus) (Bai He, 30 g), and the dried seeds of *Ziziphi Spinosae Semen* (Rhamnaceae; *Ziziphus jujuba* Mill. var. spinosa [Bunge] Hu ex H.F.Chou) (Suan Zao Ren, 30 g). PCF granules, purchased from Beijing Pharmaceutical Co., Ltd., were made of the above four botanical drugs by the process of water heating, extraction, separation, concentration, drying, and granulation. The process was operated according to the “Technical Requirements for Quality Control and Standard Formulation of Chinese Medicine Granule,” issued by the National Medical Products Administration. One dosage of PCF granule was dissolved in 100 ml distilled water which was heated at a temperature of 100°C. According to the long-term clinical practice, the adult daily dosage of PCF was 1 ml (PCF solution)/600 g (body weight)/d. The optimal dosage of the PCF solution for rats was six times greater than the adult dosage based on the body surface areas in accordance with the Chinese Medicine Pharmacology Research Technology. Furthermore, the converted lavage dose has been applied and verified on the efficacy for depression post-AMI in previous experiments (Wang et al., 2019). Thus, the optimal lavage dose was 1 ml/100 g/d.

### 2.2 Drugs and Reagents

Paquinimod (Apexbio, Houston, TX, United States), also called ABR-215757 (a specific inhibitor of S100A9), binds to S100A9 in a Ca^2+^/Zn^2+^ dependent way and blocks interaction with TLR4 (Björk et al., 2009; Liang et al., 2019). It has been applied to the experimental research of depression, atherosclerosis, and other diseases (Stenström et al., 2016; Kraakman et al., 2017). According to the literature and the results of previous studies, ABR-215757 was successively dissolved in 10% DMSO, 40% polyethylene glycol 400 (PEG400), 5% Tween 80, and 45% normal saline, and injected intraperitoneally at a dose of 5 mg/kg/d, once a day for 5 consecutive days (Masouris et al., 2017; Tahvili et al., 2018). Recombinant protein S100A9 (Bio-Techne, Minnesota, MN, United States) was prepared into 300 μg/ml mother solution with sterile water and then diluted to 0.01 μM, 0.02 μM, 0.05 μM, and 0.1 μM with a complete medium in the initial experiments. A concentration of 0.1 μM was adopted in the subsequent experiments. C34 (Apexbio, Houston, TX, United States) inhibited toll-like receptor 4 *in vitro*, which was dissolved in DMSO to prepare mother liquor with a concentration of 10 mM and diluted to the final concentration of 10 μM when used (Adegoke et al., 2019). Ferulic acid (Yuanye Bio-Technology, Shanghai, China) was dissolved in DMSO and configured to a concentration of 80 μM.

### 2.3 *In Vitro* Study

#### 2.3.1 Preparation of Medicated Sera

The rats were given PCF or distilled water as above and anesthetized by intraperitoneal injection of pentobarbital 1 hour after administration. Blood was collected from the abdominal aorta and centrifuged, heat-inactivated at 56°C for 30 min, and filtered by a 0.22 μm filter membrane. The serum was packed separately and frozen at −80°C until use.

#### 2.3.2 Cell Culture

The microglial cell line BV-2 was received from the Scientific Research Center of Shanghai 10th People’s Hospital and cultured in high-glucose Dulbecco’s-modified eagle’s medium (H-DMEM, Invitrogen, United States) with 10% fetal bovine serum (FBS Gibco, United States) and 1% penicillin/streptomycin (Gibco, United States) at 37°C in a humidified atmosphere with 5% CO2. Cells at 80% confluency were supplied for experimental treatments or trypsinized for passage.

#### 2.3.3 Group Design

BV2 microglia cells were divided into six groups: control, recombinant protein S100A9 (S100A9), C34, PCF serum (PCF), ferulic acid (FA), and control serum (CS). Except for the control group treated with complete medium, cells in the C34, FA, PCF, and CS groups were cultured in 0.1 μM of recombinant S100A9 protein for 6 h, followed by complete medium, respectively, supplemented with 10 μM C34, 80 μM FA, 5% PCF serum, and 5% control serum for 6 h.

#### 2.3.4 CCK-8 Assay

Cells were cultured in 96-well plates (2 ×10^4^ cells per well) with 100 µl complete medium containing various doses of recombinant S100A9 protein (0.01/0.02/0.05/0.1 μM), control serum (5%, 10%, 20%), or PCF serum (5%, 10%, 20%), to determine the dose-dependent effects of reagents. Cell viability was measured *via* the CCK-8 assay kits. The absorbance at 450 nm was measured with a microplate reader.

#### 2.3.5 Enzyme-Linked Immunosorbent Assay

The cell supernatants from each sample were collected for ELISA assays. Concentrations of inflammation markers, including S100A9, TNF-α, and IL-1β, were determined by pre-coated ELISA kits (MLBIO, Shanghai, China) according to the manufacturer’s instructions.

### 2.4 *In Vivo* Experiment

#### 2.4.1 Animals

Male Sprague-Dawley (SD) rats (220 ± 20 g) were purchased from Beijing Vital River Laboratory Animal Technology Co., Ltd., [License No. SCXK (Beijing) 2016–0006]. All rats were fed in a specific pathogen-free facility with controlled temperature (22 ± 1°C), relative humidity (65–70%), and a 12:12 light/dark cycle.

#### 2.4.2 Ethics

Experiments were in accordance with protocols approved by the Institutional Animal Care and Use Committee of the University of Chinese Medicine, Beijing, China (Ethical number: BUCM-4-2020091108-3141).

#### 2.4.3 Establishment of AMI Rat Model

As described previously, ligation of the left anterior descending (LAD) coronary artery was used to construct the AMI rat model, while only threading was operated without knotting in the sham group (Wang et al., 2019; Hou et al., 2021). Penicillin was injected intraperitoneally to prevent infection. The success of the AMI model was marked of pathological Q wave by more than six leads of ECG I, AVL, and V1∼V6 in electrocardiograph on the second day after surgery.

#### 2.4.4 Design and Allocation

After 7 days of acclimatization, the rats were randomly divided into the sham group (*n* = 16), AMI group (*n* = 18), PCF group (*n* = 18), and ABR-215757 group (*n* = 18). All the rats received LAD operation other than the sham group. Rats in the PCF group were administered intragastrically PCF solution (1 ml/100 g/d) at 8 a.m. every day for 5 days, while the rats in the other groups received the same volume of distilled water (1 ml/100 g/d) on the same schedule. Paquinimod (5 mg/kg/d) was injected into the rats of the ABR-215757 group intraperitoneally at 8:30 a.m. every day for 5 days, and the rats in the other groups were intraperitoneally injected with the same volume of 0.9% normal saline. After the last treatment administration, the rats underwent behavioral tests and echocardiography. Then, half of the rats randomly selected in each group were given a peritoneal injection of 1% pentobarbital sodium, and blood samples were collected from the abdominal aorta. The hearts and brain tissues were immediately isolated and snap-frozen in liquid nitrogen. On the 21st day after surgery, the remaining rats were sacrificed to detect neurogenesis in the hippocampus and cardiac fibrosis in the myocardium.

### 2.5 Behavioral Tests

Behavioral tests were performed in a double-blinded manner and operated in a dark and quiet room, and all rats were transported to which 1 hour earlier to acclimatize. The behavior in the open field test and forced swimming test was videotaped and further analyzed by SuperMaze (Softmaze, Shanghai, China), specialized animal behavior video analysis software.

#### 2.5.1 Open Field Test (OFT)

The first step was to set up the software program. In SuperMaze, the grayscale was set as the recognition algorithm and three points were determined to track the position of the rats. The open field is a square wooden chest (100 × 100 × 60 cm) with a black floor and divided into 25 identical areas with white lines. A single rat was placed in the central square and allowed to move freely for 5 min. The number of verticalities (times of rat stood on its hind limbs) was recorded by an observer blind to the group, while the total distance and distance in the central region were recorded in the software. The field was wiped clean with 75% alcohol before each test.

#### 2.5.2 Forced Swimming Test

The dynamic background method was selected, and the rats were located by the center of gravity in SuperMaze. The FST was operated in a transparent glass cylindrical tank with 60 cm in height, 38 cm in width, and 40 cm in depth. Rats were put into the glass tank filled with 22°C–24°C fresh water and allowed to swim freely for 5 min. The immobility time was recorded by the video camera and analyzed by SuperMaze software.

### 2.6 Echocardiography

The rats were anesthetized and fixed on a board with fur shaved. Three continuous cardiac cycles were captured from the left ventricular short axial section to detect the M-shaped curve. Left ventricular ejection fraction (LVEF) and left ventricular fractional shortening (LVFS) were measured to assess cardiac function. The left ventricular end-diastolic inner diameter (LViDd), left ventricular end-systolic inner diameter (LViDs), left ventricular end-diastolic volume (LVEDV), and left ventricular end-systolic volume (LVESV) were measured to evaluate the ventricular structure.

### 2.7 H&E and Masson Staining

The tissues extracted were embedded in paraffin and cut at a 4 μm thickness after fixation in 10% neutral formalin for 72 h. These slices were stained with hematoxylin/eosin (H&E) or Masson trichrome and observed under an optical microscope (Carl Zeiss Microscopy, Germany) to evaluate histopathological changes and collagen deposition. The percentage of collagen deposition area was analyzed by the ratio of fibrosis area to the total myocardial area.

### 2.8 Nissl Staining

The brains were dyed with toluidine blue O to assess neurogenesis. Brain sections were immersed in xylene and then rehydrated in graded alcohol solutions and distilled water. Subsequently, tissue slices were stained with toluidine blue (Servicebio, Wuhan, China) for 10 min, quickly rinsed in distilled water, dried at a 60°C environment, made transparent by xylene, and sealed with neutral gum. Three sample sections were selected from each group and observed using an optical microscope. The mean integrated optical density (IOD) of the dentate gyrus (DG) region in the hippocampus was measured by Image-Pro Plus 6.0 software (Media Cybernetics Inc., Rockville, MD, United States).

### 2.9 TUNEL Assay

Cardiac cell death was evaluated utilizing a TdT-mediated dUTP nick end labeling (TUNEL) assay kit (Roche, United States) in accordance with the manufacturer’s protocol. The kit was labeled with FITC fluorescein, and the positive apoptotic nucleus was dyed green. The cells were stained with DAPI (1:30, Beyotime Biotechnology, China) for nuclear counterstaining and observed under a fluorescence microscope (Zeiss Axio Scope A1). Three fields of each slice were selected for quantification. ImageJ software (NIH, MD, United States) was applied to calculate the number of TUNEL positive cells. Apoptosis index (AI) = (number of apoptotic nucleus/number of total nucleus) × 100%.

### 2.10 Immunofluorescence Staining

Paraffin sections of heart and brain tissue were processed as previously described. After routine dewaxing, hydration, and antigen retrieval, the tissues were incubated in bovine serum albumin (BSA) for 30 min. After blocking, the slices were incubated with an anti-Iba1 (1:500, Abcam, United Kingdom), anti-CD68 (1:200, Abcam, United Kingdom), or anti-S100A9 (1:500, Proteintech, United States) overnight at 4°C, followed by secondary antibodies conjugated to CY3 (1:300, Servicebio, China) or HRP (1:500, Servicebio, China). As for anti-S100A9, the slices were incubated with FITC at room temperature in the dark for 10 min. Subsequently, the tissues were stained with DAPI for nuclear counterstaining. The stained slides were photographed under a fluorescence microscope. The number of CD68^+^ cells in the myocardium or Iba1^+^ cells in the hippocampus was counted by ImageJ software (NIH, MD, United States) in a blinded manner. The data were expressed as the mean number of cells per square millimeter. For intensity measurements, three sections from each sample at the same level were used to determine the mean optical density (mean optical density = IOD/area). The mean values were calculated from three randomly selected microscopic fields from each section.

### 2.11 Western Blotting

The hippocampal and myocardial samples were lysed; then, proteins were extracted with RIPA buffer (Thermo Fisher Scientific, United States) and measured by the BCA protein concentration Determination kit (Glpbio, United States). Protein mixtures were separated *via* 10% SDS-PAGE and transferred to polyvinylidene difluoride (PVDF) membranes (Millipore, United States). TBST containing non-fat dried milk was used to block non-specific binding to the membranes, and the membranes were incubated with primary antibodies at 4°C overnight, followed by incubation with the secondary antibodies (1:3000, Thermo Fisher Scientific) at room temperature for 30 min and reaction with enhanced chemiluminescence (ECL). The primary antibodies for immunoblotting were as follows: anti-TLR4 (1:1000, Abcam), anti-NF-κB (1:1000, Abcam), anti-BDNF (1:1000, Abcam), anti-GAPDH (1:1000, Servicebio), and anti-ACTIN (1:1000, Servicebio). The exposure condition was adjusted on the basis of luminescence intensity. The results were scanned and color-modulated, and the target band intensities were analyzed by the BandScan software (Glyko, United States).

### 2.12 Real-Time Quantitative PCR

TRIzol and chloroform were used to extract mRNA from tissues and cells. Purity was assessed by the ratio of A260/A280, and RNA with a purity between 1.8 and 2.0 was used for the next actions. The complementary strand DNA was synthesized from RNA *via* first-strand cDNA synthesis mix with gDNA Remover F0201-100T Kit (LABLEAD, China). The real-time PCR reaction system was formulated as requested by QuantiNova SYBR Green PCR Kit (QIAGEN, Germany). Each reaction was run in 35–40 cycles consisting of the following steps: initial heat activation at 95°C for 2 min followed by a set cycle of denaturation at 95°C for 5 s and combined annealing/extension at 60°C for 10 s. Melt curve analysis was performed to confirm the specificity of the amplicon. As a final step, relative mRNA expression levels were analyzed using the formula ΔΔCt method and normalized to the GAPDH.

### 2.13 Sequences of PCR Primers

**Table:** 

Gene symbol	Forward	Reverse
S100A9	GACATCCTGACACCCTGAACAAG	CCCATCAGCATCATACACTCCTC
NF-κB	TTATGGGCAGGATGGACCTA	CTCCTTCGGAACGATATGAT
GAPDH	CTGGAGAAACCTGCCAAGTATG	GGTGGAAGAATGGGAGTTGCT

### 2.14 LC–MS/MS Method

The hippocampus tissue samples were weighed and ground in the frozen grinding machine. 80% methanol was added at a ratio of 1:10, followed by vortex, low-temperature ultrasound for 10 min, and centrifugation at 13000 rpm for 10 min. Then, the supernatant was removed to frozen centrifugation and concentrated to dry, and 100 ul of solvent was added for redissolution. The analysis was performed on the AB SCIEX QTRAP 4500 (United States) triple quadrupole mass spectrometer in SRM and positive ionization mode. The LC separation was run on an ACQUITY HSS PFP column (2.1 × 100 mm, 1.7 μm, United States) equipped with Waters ACQUITY UPLC I-Class infinite binary pump. Acetonitrile containing 10 mM amine acetate and 0.1% formic acid was used as solvent A, and water containing 10 mM amine acetate and 0.1% formic acid was used as solvent B. The flow rate was 0.2 ml/min. The steps of gradient elution were as follows: the initial conditions were 98% solvent B starting from 0 min, 8 min to 0% solvent B, returning to the initial state of 98% solvent B after 2 min, and 12 min to end a collection. The column temperature was 35°C, while the sample was kept at 10°C and the injection volume was 10 μl. The MS parameters were as follows: ESI ion source temperature, 500°C; air curtain, 30 psi; collision activated dissociation gas settings, medium; and ion spray voltage, 5500 V. All data were processed by Analyst 1.6.3 Software.

### 2.15 Statistical Analysis

The data were presented as mean ± standard deviation (SD). Statistical graphing was performed using GraphPad Prism software (version 8.0; Inc., San Diego, CA, United States). For multiple comparison tests, one-way analysis of variance (ANOVA) was performed, followed by a Tukey *post hoc* test. Data were analyzed for normality and homogeneity of variances as a justification for parametric or nonparametric analyses. For all analyses, an average value of *p* < 0.05 was considered statistically significant.

## 3 Results

### 3.1 PCF Improved Cardiac Function and Ventricular Remodeling in AMI Rats

**FIGURE 1:**
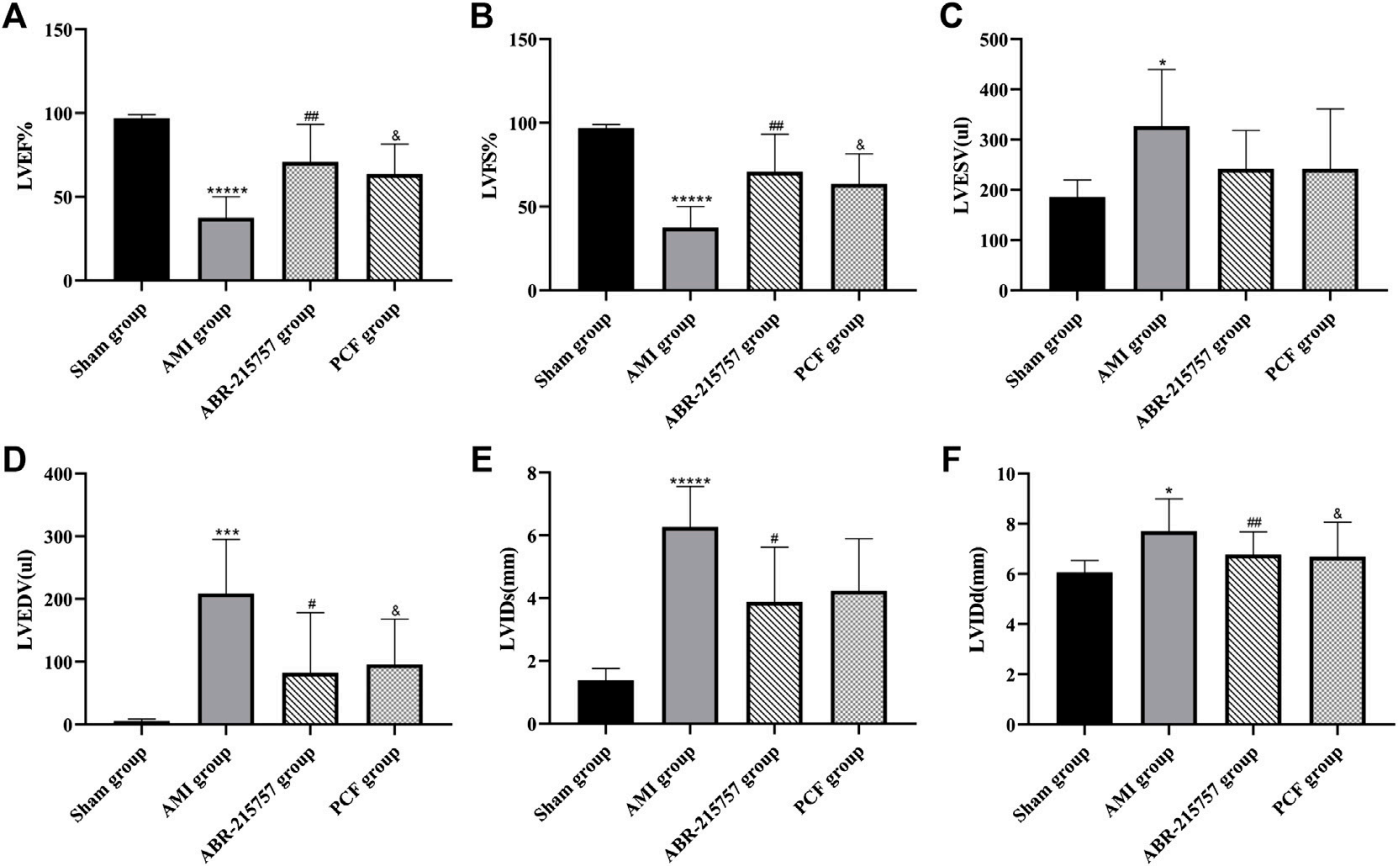
Cardiac function parameters of rats after myocardial infarction in echocardiography from each group (*n* = 7). Values were expressed as mean ± SD. LVEF: left ventricular ejection fraction. LVFS: left ventricular fractional shortening. LViDd: left ventricular end-diastolic inner diameter; LViDs: left ventricular end-systolic inner diameter. LVEDV: left ventricular end-diastolic volume. LVESV: left ventricular end-systolic volume. *p < 0.05, ***p < 0.001, *****p < 0.00001, compared with the sham group. #*p* < 0.05, ##*p* < 0.01, compared with the AMI group. & *p* < 0.05, compared with the AMI group.

As shown in Figure 1, myocardial infarction led to wall thinning, dilated left ventricular chambers, and an obvious decrease in cardiac function. The LVEF and LVFS were decreased in the AMI groups compared with the sham group (*p* < 0.00001). In contrast, the LVEF and LVFS in the PCF group were significantly elevated compared with the AMI group (*p* < 0.05) and showed a similar trend in the ABR-215757 group (*p* < 0.01). Thus, PCF and ABR-215757 could overcome the inhibitory effects of AMI on the LVEF and LVFS. The LVIDd and LVIDs, respectively, indicated end-diastolic and end-systolic left ventricle internal dimension, while LVESV and LVEDV, respectively, showed the maximum volume of the ventricle in systole and diastole. The four indicators in the PCF group and ABR-215757 group were declined in contrast with the AMI group.

### 3.2 PCF Alleviated Histological Injury in Myocardial Tissue of AMI Rats

**FIGURE 2:**
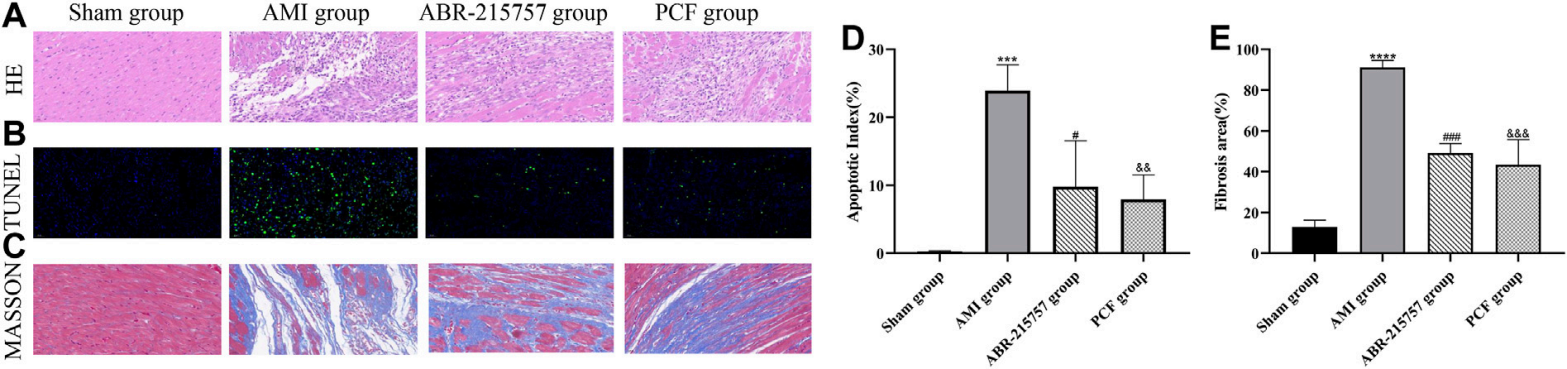
Effect of PCF on AMI-induced pathological changes in the myocardial tissue. **(A)** H&E staining showed different levels of inflammatory infiltration in the peri-infarct border zone. Scale bar, 40 μm. **(B)** TUNEL staining showed cardiac apoptosis on 7 days after MI surgery. Green staining of the nucleus indicated apoptosis, and blue staining marked DAPI. **(C)** Masson trichrome staining of heart slides at 21 days after MI. Red, myocardium; blue, scarred fibrosis. **(D)** Quantitative analysis of TUNEL-positive cells in the border zone of infarction area. Three separate fields were calculated from each group. **(E)** Fibrosis area as a percentage (three samples from each group). ***p < 0.001, ****p < 0.0001, compared with the sham group. #*p* < 0.05, ###*p* < 0.001, compared with the AMI group. && *p* < 0.01, &&& *p* < 0.001, compared with the AMI group.

The severity of cardiac damage was evaluated by morphological observations (Figure 2). Hematoxylin/eosin staining showed an orderly arrangement of myocardial fibers in the sham group. Conversely, the myocardial fibers became loosely and irregularly arranged in the AMI group. Instead, PCF and ABR-215757 alleviated the morphological injuries after AMI. Compared with the sham group, the apoptosis index was significantly increased in the AMI group on day 7 after coronary ligation (*p* < 0.001); then, a large number of fibrotic scars were observed on day 21 (*p* < 0.0001). TUNEL assay revealed that PCF and ABR-215757 significantly ameliorated AMI-induced cell apoptosis (Figure 2D), and Masson staining showed that both of them significantly decreased the fibrosis area in the peri-infarct border zone (*p* < 0.001, Figure 2E), indicating their beneficial effects to reduce impairment of cardiac function.

### 3.3 PCF Improved Depression in Rats After AMI

**FIGURE 3:**
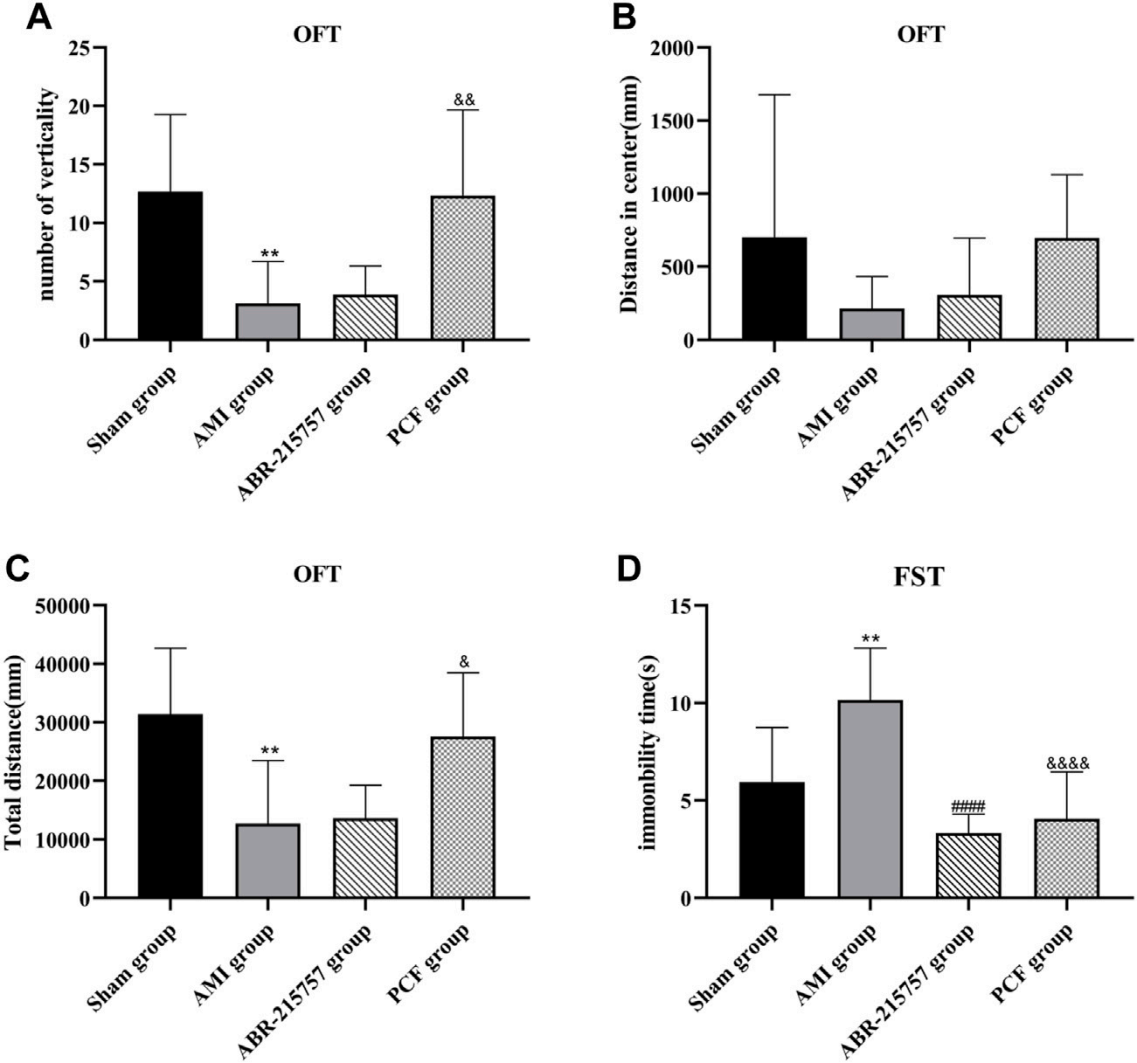
PCF significantly ameliorated depression-like behavior in rats after myocardial infarction. **(A)** The number of verticality in open field test (OFT). **(B)** Distance in center. **(C)** Total distance. **(D)** Immobility time of rats in forced swimming test (FST). *n* = 9 per group. **p < 0.01, compared with the sham group. ####*p* < 0.0001, compared with the AMI group. & *p* < 0.05, && *p* < 0.01, &&&& *p* < 0.0001, compared with the AMI group.

Compared with the sham group, rats in the AMI groups showed depression-like behaviors, such as a reduction of crossing zones and rearing times in OFT and longer immobility time in FST (*p* < 0.01, Figure 3). In contrast, rats in the PCF group were much more active, such as extended total distance (*p* < 0.05) and increased verticality number (*p* < 0.01) in OFT and much shorter immobility duration in FST (*p* < 0.0001). Interestingly, rats after intraperitoneal injection of paquinimod showed less horizontal movement and fewer rearing times in OFT (*p* > 0.05) but shorter immobility time in FST (*p* < 0.0001).

**FIGURE 4:**
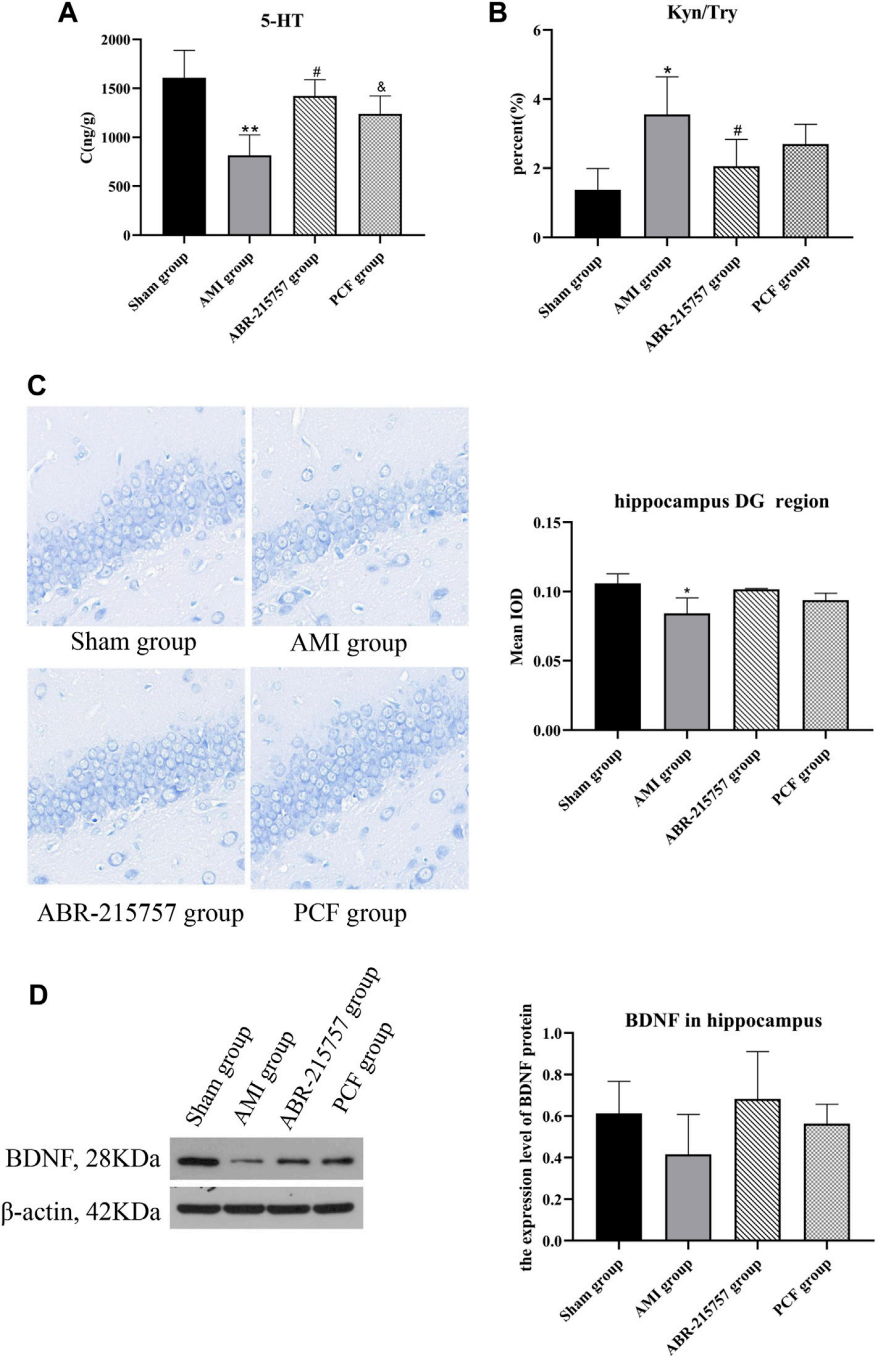
The effect of PCF on the neurotransmitter disorder and decreased neurogenesis caused by myocardial infarction. **(A)** HPLC analysis of 5-HT in the hippocampus of rats (*n* = 3). **(B)** Ratio of kynurenine to tryptophan. **(C)** Nissl’s staining in the dentate gyrus region of hippocampus from different groups. **(D)** Western blotting detected the protein expression levels of BDNF in the hippocampus (*n* = 3). 5-HT: 5-hydroxytryptamine; Kyn: kynurenine; Try: tryptophan; BDNF: brain-derived neurotrophic factor; DG: dentate gyrus. *p < 0.05, **p < 0.01, compared with the sham group. #*p* < 0.05, compared with the AMI group. & *p* < 0.05, compared with the AMI group.

The mainstay of antidepressant therapy directs at serotonin (5-hydroxytryptamine, 5-HT) metabolism, in detail, blocking the reuptake of 5-HT from the extracellular space. As a precursor for serotonin, 95% of tryptophan is degraded in the liver through the kynurenine pathway, and the remaining is used for the synthesis of 5-HT (Oxenkrug, 2013). Abnormalities in the tryptophan-kynurenine pathway are implicated in the pathophysiology of depressive disorder (Muneer, 2020). To examine the effects of AMI on neurotransmitters in the brain, we analyzed the hippocampus tissues by liquid chromatography-mass spectrometry (LC-MS). As shown in Figures 4A, significant decrease in 5-HT level was observed in AMI rats (*p* < 0.01), while PCF and ABR-215757 treatment led to a degree of recovery (*p* < 0.05). The level of tryptophan (Try) was altered in a manner similar to that of 5-HT. Proinflammatory cytokines catalyze the conversion of Try to kynurenine (Kyn), and the kynurenine pathway may elucidate the phenomenon of inflammation in depression (Vancassel et al., 2018). It has evoked widespread concern that the ratio of kynurenine to tryptophan is significantly enhanced in patients with depression (Maes et al., 2002; Zhang et al., 2016). Our results showed an increased hippocampal Kyn/Try ratio in the AMI group (*p* < 0.05), and the ratio declined with the administration of S100A9 inhibitors (*p* < 0.05, Figure 4B).

Depression is associated with neuroplasticity in the brain regions, particularly the hippocampus. Nissl bodies, easily stained by toluidine blue, reflects the synthesis of Nissl bodies and the survival of nerve cells. Nissl staining revealed that the rats had fewer neurons with the loose arrangement in the hippocampal DG regions on day 21 after coronary artery ligation (*p* < 0.05, Figure 4C), whereas no obvious hippocampal neuron loss was observed in the PCF group and ABR-215757 group. Brain-derived neurotrophic factor (BDNF), a topic neurotrophic factor of intensive research in the mammalian brain, contributing to the maintenance and survival of neurons and activity-dependent regulation of synapse number and function, is integral to the pathophysiology of depression (Zhang et al., 2016). Multiple lines of evidence implied that administration of BDNF into either hippocampus or midbrain in rodent models produces an antidepressant-like effect (Monteggia et al., 2007). In this study, the expression level of BDNF was downregulated in the AMI group compared with the sham group and showed an upward trend after administration of PCF and ABR-215757, but there was no statistical significance between groups (Figure 4D).

### 3.4 PCF Inhibited the Activation of S100A9/TLR4/NF-κB Signaling Pathway

**FIGURE 5:**
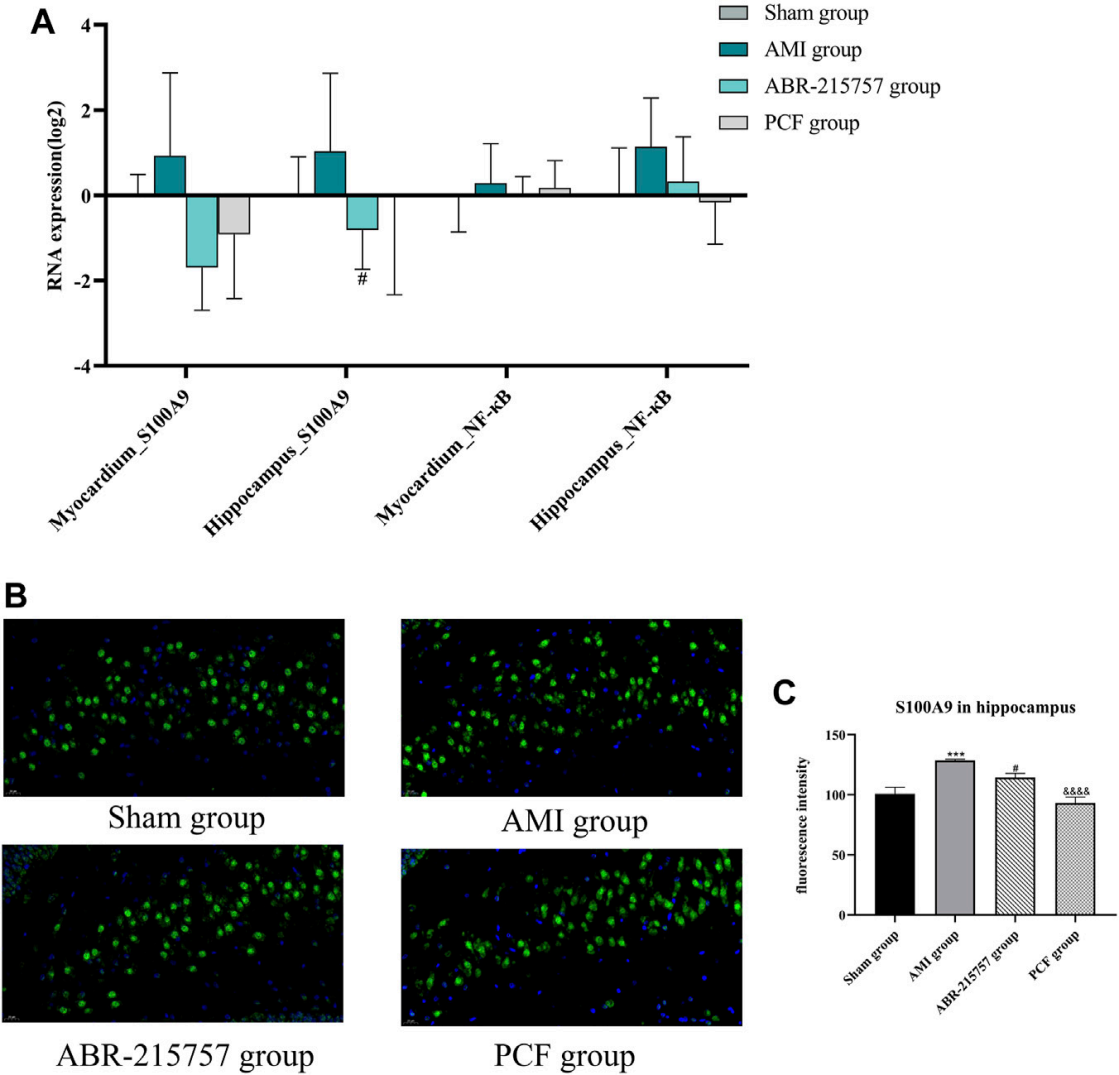
The effect of PCF on the S100A9/NF-κB pathway in the hippocampus and myocardium. **(A)** The gene expression of S100A9 and NF-κB in the hippocampus and myocardium detected by RT-qPCR. Data were presented as mean ± SD, *n* = 3 per group. **(B)** Immunofluorescence analysis of S100A9 in hippocampus tissues. S100A9 immunostaining was shown in green and DAPI in blue. **(C)** Mean optical density of S100A9-positive cells. Three fields were selected from each slide. ***p < 0.001, compared with the sham group. #*p* < 0.05, compared with the AMI group. &&&& *p* < 0.0001, compared with the AMI group.

**FIGURE 6:**
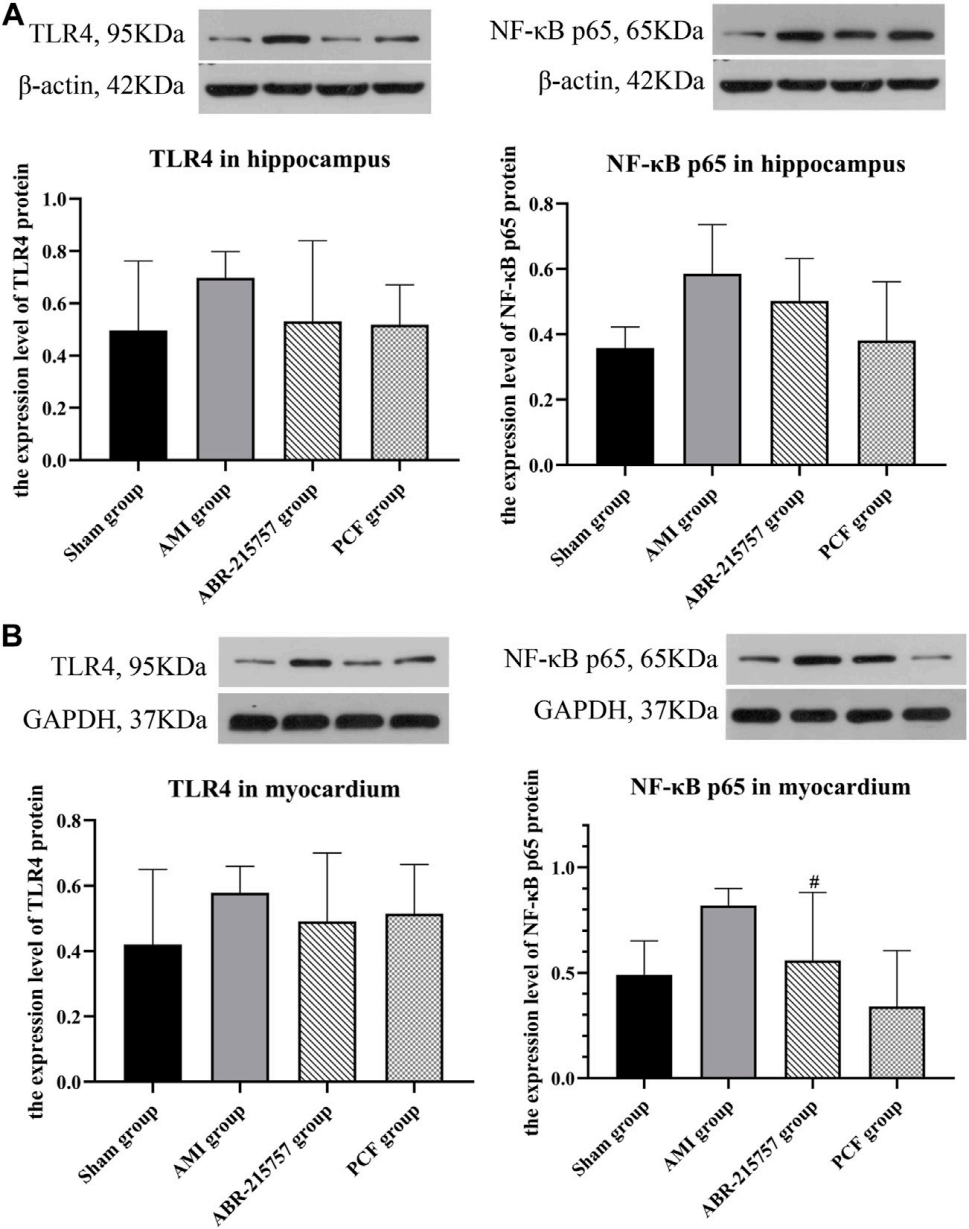
Protein expression of TLR4 and NF-κB detected by western blotting. **(A)** The protein bands of TLR4 and NF-κB in the hippocampus. β-actin was reused as the loading control for protein TLR4 and NF-κB p65. **(B)** The protein bands of TLR4 and NF-κB in the myocardium. Data were presented as mean ± SD. *n* = 3 per group. #*p* < 0.05, compared with the AMI group.

Our present proteomic study revealed that S100A9 was the only molecule intersected from numerous proteins in the myocardium and hippocampus and one of the differentially expressed proteins among the sham, AMI, and PCF groups. We first verified the *in vivo* effect of coronary ligation on the S100A9 expression by RT-qPCR. Consistent with proteomic data, the expression level of S100A9 gene was elevated in the AMI group compared with the sham group and was returned to the basal level by PCF treatment (Figure 5A). The expression of S100A9 protein in the hippocampus was also visualized by immunofluorescence. Expression analysis showed increased expression of S100A9 in the AMI group (*p* < 0.001), and S100A9-positive fluorescence intensity were markedly decreased in the PCF group (*p* < 0.0001) and ABR-215757 group (*p* < 0.05, Figure 5B). In addition, the immunoblotting analysis showed that the expression of TLR4 and NF-κB protein was changed in a manner similar to that of S100A9 (Figure 6). Interestingly, the protein expression trend was observed in the myocardium and hippocampus. In order to explore the effect of S100A9 on inflammatory factors, the ABR-215757 group was set and the expression pattern was found to be parallel to that in the PCF group. As shown in Figure 5A and Figure 6A, compared with the AMI group, the expression of the S100A9 gene in the hippocampus and NF-κB protein in the myocardium from the ABR-215757 group was downregulated (*p* < 0.05). However, it is regrettable that there was no statistically significant difference in protein expression between the PCF and AMI group.

### 3.5 PCF Reduced the Contents of Proinflammatory Factor

**FIGURE 7:**
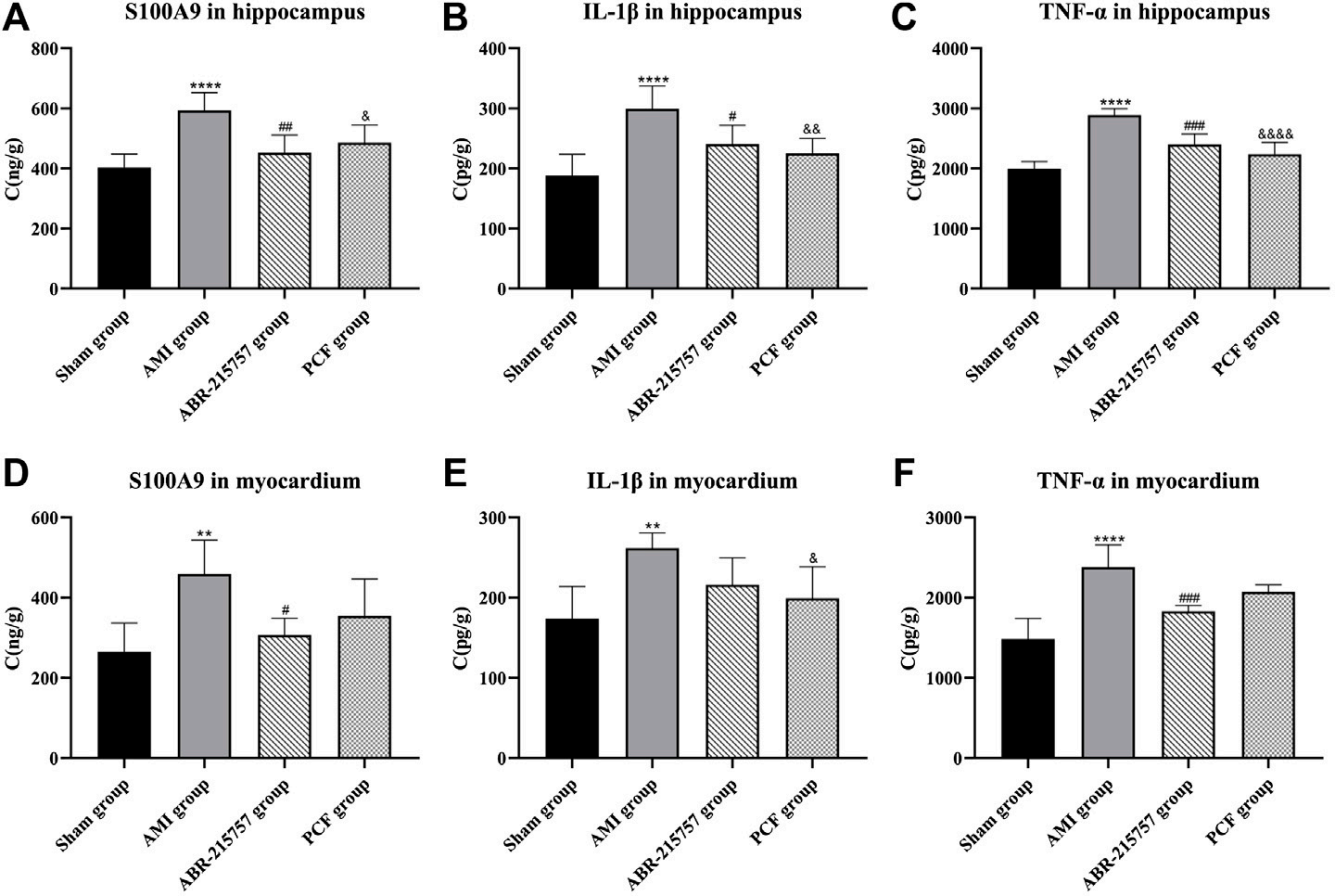
Levels of S100A9, IL-1β, and TNF-α in the hippocampus and myocardium were determined by an ELISA assay. Values were expressed as mean ± SD. *n* = 6 per group. **p < 0.01, ****p < 0.0001 compared with the sham group. #*p* < 0.05, ##*p* < 0.01, ###*p* < 0.001, compared with the AMI group. & *p* < 0.05, && *p* < 0.01, &&&& *p* < 0.0001, compared with the AMI group.

Furthermore, we detected the expression of inflammatory cytokines (S100A9, IL-1β, and TNF-α) in the hippocampus and myocardium by ELISA. As shown in Figure 7, S100A9 levels in the myocardium were significantly increased in the AMI group (*p* < 0.01), while those in the sham group remained low. In contrast with the sham group, coronary ligation not only significantly increased the IL-1β (*p* < 0.01) and TNF-α levels (*p* < 0.0001) in the myocardium but also elevated the levels of inflammatory factors in the hippocampus to promote neuroinflammation (*p* < 0.0001). On the contrary, the results showed that relative to the AMI group, the hippocampal S100A9 (*p* < 0.01), IL-1β (*p* < 0.05), and TNF-α (*p* < 0.001) levels were obviously downregulated after ABR-215757 intervention, while the myocardial S100A9 (*p* < 0.05) and TNF-α (*p* < 0.001) levels also showed a clear trend of descending. Besides, we found that PCF reduced myocardial IL-1β level compared with the AMI group (*p* < 0.05), and the levels of S100A9 (*p* < 0.05), IL-1β (*p* < 0.01), and TNF-α (*p* < 0.0001) were declined in the hippocampus.

### 3.6 PCF Inhibited the Activation of Macrophages/Microglia

**FIGURE 8:**
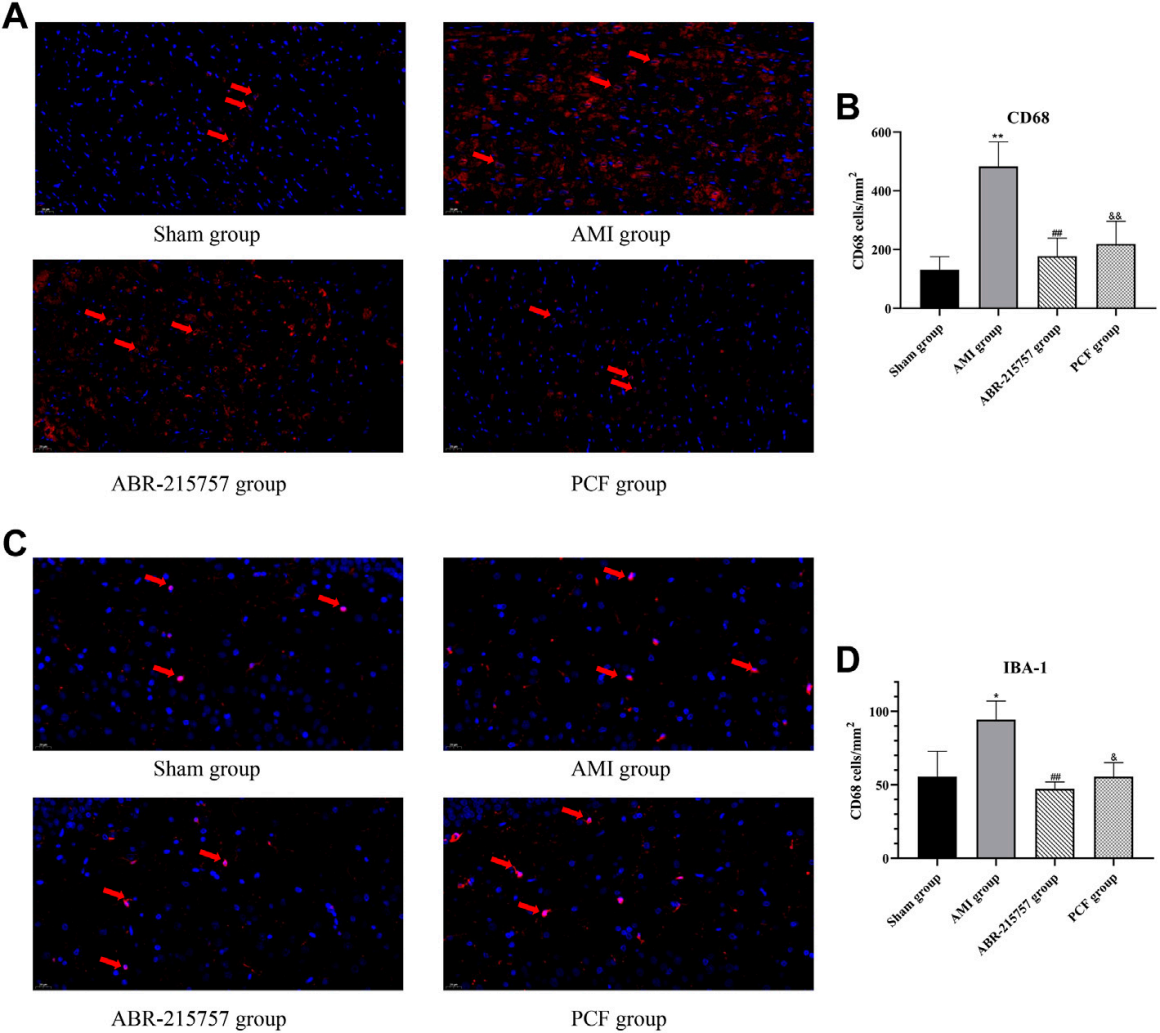
Macrophage activation in the myocardium and microglial activation in the DG region of AMI rats by immunofluorescence staining. **(A)** Representative images of CD68 positive cells in the myocardium. **(B)** Quantification of the CD68^+^ cell number. **(C)** Representative images of Iba1 positive cells in the DG region of the hippocampus. **(D)** Quantification of the Iba1^+^ cell number. *n* = 3 slices from each group. *p < 0.05, **p < 0.01, compared with the sham group. ##*p* < 0.01, compared with the AMI group. & *p* < 0.05, && *p* < 0.01, compared with the AMI group.

CD68 and Iba1 are recognized as specific markers, respectively, for macrophage and microglia. In order to investigate the effect of AMI on the activation of the macrophage in the heart, immunofluorescence staining for myocardial sections was performed. Results indicated that acute myocardial ischemia significantly increased the number of CD68 positive cells (*p* < 0.01, Figures 8A,B). However, PCF and ABR-215757 treatment inhibited macrophage activation and decreased the number of CD68^+^ cells (*p* < 0.01). We further interrogated the effect of cardiac surgery on hippocampal microglia. As the results indicated, hippocampal microglia were activated by coronary ligation, while PCF and ABR-215757 treatment decreased the number of Iba1^+^ cells in the hippocampal region (*p* < 0.01 for ABR-215757, *p* < 0.05 for PCF, Figures 8C,D).

### 3.7 The Effect of Recombinant Protein S100A9 on the Viability of BV2 Cells

**FIGURE 9:**
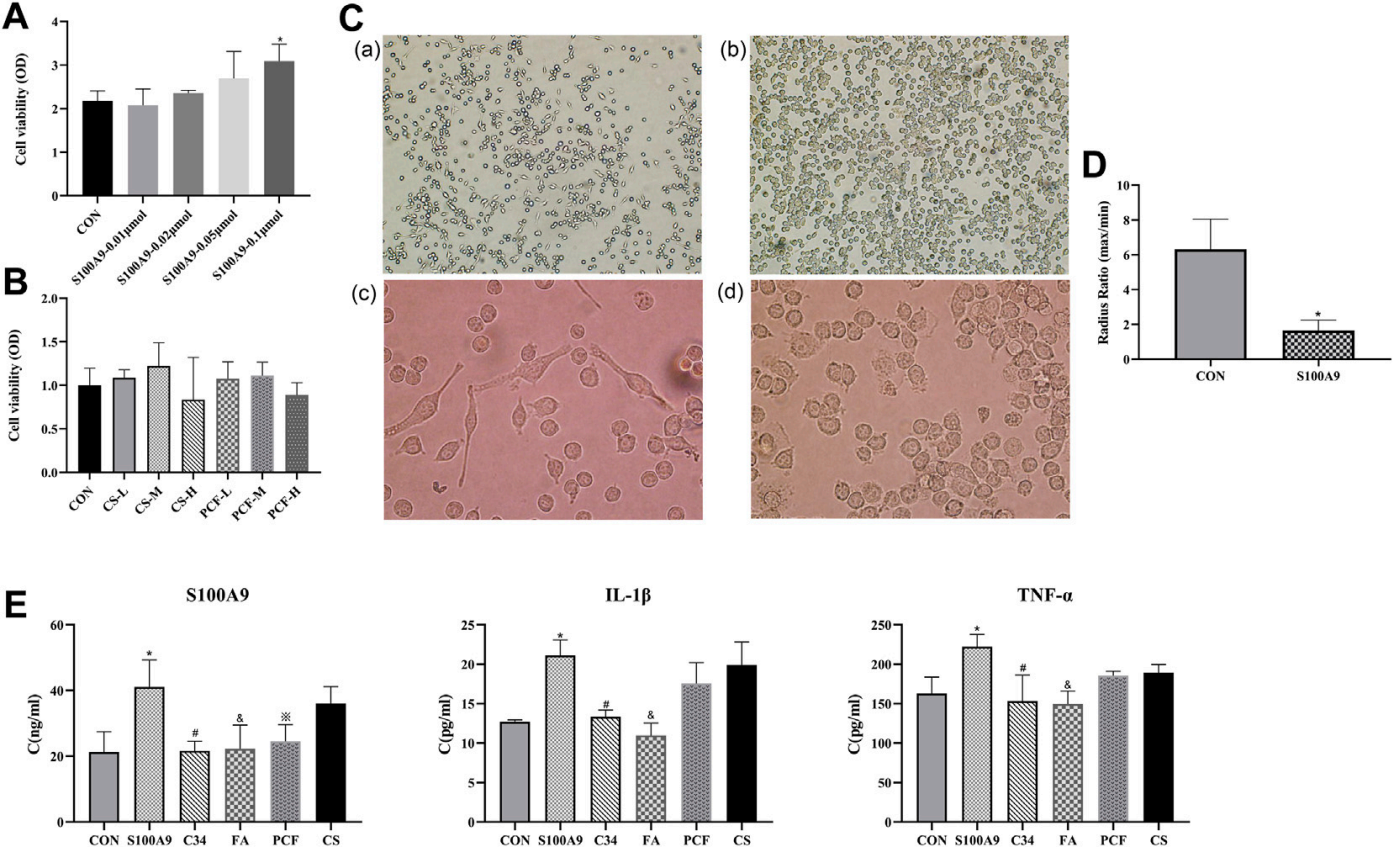
PCF serum reduced the proinflammatory effect of recombinant protein S100A9 in BV2 cells *via* inhibiting TLR4. **(A)** Microglia cells were treated with protein S100A9 (0.01,0.02, 0.05, 0.1 μmol) for 6 h. CCK-8 assay was used to detect the viability of BV2 cells. **(B)** Effect of PCF serum with a concentration of 5%, 10%, and 20% on the viability of BV-2 cells. **(C)** Resting microglia (a, ×100 magnification; c, ×400 magnification) and activated microglia (b, ×100 magnification; d, ×400 magnification). Morphological changes of BV2 microglia after incubation with S100A9 protein for 6 h (b,d). **(D)** The ratio of the maximum radius to the minimum radius in BV2 microglia. **(E)** The level of S100A9, IL-1β, and TNF-α in the culture media was measured with ELISA. Each value represented the mean ± SD of three independent experiments. CS: control serum; CS-L: control serum low dose; CS-M: control serum medium dose; CS-H: control serum high dose; PCF: PCF serum; PCF-L: PCF serum low dose; PCF-M: PCF serum medium dose; PCF-H: PCF serum high dose; C34: TLR4 inhibitor; FA: ferulic acid; *p < 0.05, compared with the control group. #*p <* 0.05, compared with the S100A9 group. & *p <* 0.05, compared with the S100A9 group. ※ *p <* 0.05, compared with the S100A9 group.

CCK-8 assay was applied to determine the effect of recombinant protein S100A9 on the viability of BV2 cells. As shown in Figure 9A, the administration of protein S100A9 with 0.01 μmol–0.05 μmol for 6 h had no significant effect on the viability of microglia cells, while 0.1 μmol of S100A9 could observably promote microglial cell proliferation (*p* < 0.05). Therefore, 0.1 μmol of S100A9 was utilized in the following experiments.

### 3.8 S100A9 Induced Morphological Changes of Microglia Cells

As “sentinels” of the nervous system, it is fitting that microglia respond to changes in biological signaling. Further investigation in BV2 cells observed two major morphological phenotypes, amoeboid *versus* ramified (Figure 9C). Resting microglia cells existed mostly with oblate bodies, as well as stretched and elongated synapses. Under the activation of recombinant S100A9 protein, microglia cells became enlarged, retracted their processes, formed new motile protrusions, and transformed into spherical or ameboid form. Parameters such as cell area and radius can be used to describe the microglia activation state. Then, a more detailed morphological characterization was carried out. The results revealed that compared with the control serum group, S100A9 induced decreases in radius ratio (*p <* 0.05, Figure 9D). These morphological modulations indicated that the activation of microglia was attributed to the S100A9 stimulation.

### 3.9 Effect of PCF Serum on S100A9-Induced Activation of Inflammatory Factors

This study investigated whether PCF serum was involved in the suppression of the inflammatory response induced by S100A9. As shown in Figure 9E, inflammatory factors including S100A9, TNF-α, and IL-1β were markedly increased following treatment with the recombinant S100A9 protein, compared with those in the control group (*p* < 0.05). No marked reduction in inflammatory factors was observed in the control serum group. However, treatment with PCF serum resulted in a significant reduction in protein S100A9 (*p* < 0.05). There might be viewpoints that components cannot be identified in drug serum, which represents a “black box operation” with unclear pharmacodynamic substance. Indeed, we have previously detected pharmaceutical ingredients in serum from rats intragastrically by PCF utilizing LC-MS and found that ferulic acid might be a major molecule component of PCF. Therefore, the inhibitory effect of ferulic acid on S100A9-induced inflammatory factors was also examined. The result turned out that ferulic acid could not only reduce S100A9 content (*p* < 0.05) but also inhibit the expression of TNF-α and IL-1β (*p* < 0.05). In addition, C34 (TLR4 inhibitor) was proved to reverse the proinflammatory effects of S100A9 (*p* < 0.05).

### 3.10 Effect of PCF Serum on the Viability of BV-2 Cells

In order to confirm that the anti-inflammatory property of PCF serum was not due to cytotoxic effects on the BV-2 microglial cells, the drug serum group was further divided into three subgroups with a concentration of 5%, 10%, and 20%, respectively. As can be seen from Figure 9B, the viability of the BV-2 cells was not reduced following treatment with low and medium concentrations of PCF. BV-2 cell viability was slightly decreased in the high dose group, but there was no statistical significance compared with the control group. These results indicated that the inhibitory effects of drug serum on the S100A9-induced inflammatory response did not result from its cytotoxic action.

## 4 Discussion

Related proteins and immunoinflammatory phenotypes predicted by previous proteomics were examined in this study. Our research highlighted that PCF inhibited macrophage/microglia inflammation by the suppression of S100A9 signaling after AMI, thus improving cardiac function and depression-like behavior. PCF serum and ferulic acid alleviated microglia inflammation *in vitro*.

According to proteomics results from our previous studies, we speculated that PCF might regulate S100A9-mediated over-activation of macrophage/microglia inflammation, thus leading to mitigation in subsequent inflammatory processes involved in AMI. The dramatic cardiomyocyte death initiates a cascade of inflammation in AMI, in the process of which the role of alarmin S100A9 in deteriorating cardiac function has become a hot topic supported by several top journals of clinical and experimental evidence in these years (Li et al., 2019; Marinković et al., 2020; Sreejit et al., 2020). S100A9, as a potent activator of the innate immune response, as well as the damage-associated molecular pattern (DAMP) protein, is abundantly expressed in neutrophils and rapidly released from activated neutrophils, monocytes/macrophages, and dying cardiomyocytes into the coronary and systemic circulation after myocardial ischemia (Schiopu and Cotoi, 2013). S100A9 interacted locally with toll-like receptor 4 (TLR4) or receptor of advanced glycation end products (RAGE) to promote the expression of NF-κB and release IL-1β and TNF-α (Ehrchen et al., 2009; Riva et al., 2012). The regulatory role of S100A9 in macrophage activation has been brought into focus. The continuous activation of macrophages might be actuated by the S100A9 protein, which acts as a character at the center of the stage to orchestrate the functions of the individual players in cooperation with other proinflammatory cytokines (Ganta et al., 2019; Marinković et al., 2020). Stankiewicz et al. recently analyzed the hippocampal transcriptome of mice subjected to acute and chronic social stress of different durations and found that hippocampal S100A9 mRNA increased (Stankiewicz et al., 2015). In addition, central injection of recombinant S100A9 proteins could evoke depressive-like behaviors, the activation of TLR4/NF-κB signaling, and microglia. The effects of S100A9 protein were attenuated by TLR4 inhibitor TAK-242, indicating that the dysfunction of S100A9/TLR4 signaling in the hippocampus could generate neuroinflammation and depression-like behaviors (Gong et al., 2018). *In vitro* studies also showed that S100A9 observably increased the secretion of proinflammatory cytokines, including TNF-α and IL-6, in cultured BV-2 microglial cells, the process of which was suppressed by TLR4 inhibitors (Ma et al., 2017). Microglia activation is not only a hallmark of neuroinflammation but also contributes to the development of depressive-like behaviors. Recent studies demonstrated that the impairment of the normal structure and function of microglia caused by intense inflammatory activation can result in depression and associated impairments in neuroplasticity and neurogenesis. Accordingly, some forms of depression can be recognized as a microglial disease (microgliopathy) (Yirmiya et al., 2015). Hippocampal microglial activation was demonstrated to originate from stress and be implicated in the pathophysiology of depression. Thus, the hippocampus, a region with a high density of microglial cells (Brites and Fernandes, 2015), was selected to be tested instead of other brain organs. Similar to the above results, in AMI-induced depressive rats, the level of S100A9 showed an increasing trend in the myocardium and the hippocampus, accompanied by the activation of transcription factor NF-κB and the release of proinflammatory factors. Also, our research showed a higher content of S100A9 in the myocardium and the hippocampus by ELISA in the AMI group. Coronary ligation promoted the activation of macrophage/microglia, respectively evidenced by an increase in the number of myocardial CD68 positive cells and hippocampal Iba1 positive cells. Intragastric administration of PCF downregulated the expression of S100A9 and other inflammatory factors and inhibited the activation of microglia. Our results revealed that PCF intervention inhibited inflammation, which might partly attribute to a reduction in the content of S100A9 and the inhibiting effect of macrophage/microglia activation.

For additional verification of the mechanism, ABR-215757 was used to inhibit S100A9. Paquinimod exerts consistent and robust immunomodulatory effects on systemic lupus erythematosus, positively evaluated in a phase 2 randomized controlled trial (Bengtsson et al., 2012). The application range of paquinimod has gradually expanded in preclinical studies and mainly lies in its inhibition of inflammatory reaction by blocking the interaction with TLR4 and RAGE (Kraakman et al., 2017; Boros and Vécsei, 2020). Paquinimod is second-generation quinoline-3-carboxamides and may be a novel promising therapeutic way for depressive disorder (Boros and Vécsei, 2020). At the moment, *in vivo* studies demonstrate that ABR-215757 effectively ameliorates depressive symptoms (Gong et al., 2018). In our research, after continuous administration of ABR-215757 in the whole acute phase, the expression levels of S100A9, NF-κB, IL-1β, and TNF-α were significantly downregulated. Moreover, the inhibition of macrophage/microglia activation by ABR-215757 was shown to alleviate inflammation and modulate 5-HT metabolism. As a result of TLR4 signaling blocking, the desperate behavior was successfully rescued, and the cardiac function was partially restored. It is noteworthy that ABR-215757 could ameliorate depression-like behavior, characterized by improvement in despair rather than interest and exploration. Separate depressive symptoms may be encoded by differential changes in distinct circuits in the nervous system. An article published in *Cell* in 2017 reported that distinct neuronal projections to the lateral habenula and ventral tegmental area subserved different depressive behaviors: behavioral despair and social withdrawal, respectively (Knowland et al., 2017). The results of the behavioral test implied that ABR-215757 had distinct effects on different depressive phenotypes. Reviewing the related literature, we hypothesized that the separate effect of ABR-215757 on different phenotypes of depression might originate from the diverse projection of neurons, deserving further exploration.

However, it is still not clearly identified that S100A9 induced an inflammatory response *via* the TLR4 receptor nor that PCF inhibited microglial inflammation through this pathway. Therefore, we conducted cell experiments in which BV2 microglia were stimulated by recombinant S100A9 protein at a concentration of 0.1 μmol to construct a model group. Our results showed that S100A9 could induce the release of IL-1β and TNF-α in microglial cells. Cellular morphology revealed the characters of recombinant S100A9 in the activation of microglia. An increase in IL-1β and TNF-α levels derived from activated microglia may promote depressive symptoms. In addition, the C34 (TLR4 inhibitor) group and PCF (PCF serum) group were set up to elucidate the mechanisms that *in vivo* studies have failed to elucidate. The inhibition of TLR4 attenuated these effects of S100A9, indicating that S100A9-induced microglia activation depended on TLR4 signaling. We examined whether or not the expression of TNF-α and IL-1β induced by S100A9 was inhibited by the treatment with PCF serum on the BV2 microglia. The expression of inflammatory markers was significantly upregulated by S100A9 and showed a downward trend by the co-treatment with PCF serum.

In the infarcted myocardium caused by prolonged coronary occlusion, the DAMP proteins released from necrotic cells trigger both myocardial and systemic inflammatory responses. Inflammatory cells clear the infarct of dead cells and matrix debris and activate repair by myofibroblasts and vascular cells, but they may also lead to adverse fibrotic remodeling of viable segments and accentuate cardiomyocyte apoptosis (Huang and Frangogiannis, 2018). Induction of cytokines and upregulation of endothelial adhesion molecules modulate leukocyte recruitment in the infarcted heart tissues. Apoptosis, a process of programmed cell death, has been proposed to occur in response to proinflammatory cytokines after myocardial ischemia (Frangogiannis, 2015). In the present study, we measured the inflammation level and occurrence of apoptosis severally by HE staining and TUNEL staining in the heart tissues on day 7 after coronary ligation. Also, Masson staining was applied to assess myocardial fibrosis at 21 days after AMI. Severe inflammatory infiltrates, myocardial fiber rupture, increased apoptosis index, and fibrotic regions were shown in the AMI group. Conversely, these pathological phenomena were alleviated by the administration of PCF.

There is considerable evidence that behavioral impairments observed after AMI are consistent with a model of human post-MI depression (Wann et al., 2007; Bah et al., 2011a; Bah et al., 2011b). A majority of studies accorded closely with the conclusion, and Wann et al. (2007) put much effort into making it convincing (Bah et al., 2011a). Wann et al. (2007) reported that MI rats display behavioral signs compatible with depression 2 weeks after the cardiovascular event, including anhedonia (i.e., less sucrose intake) and behavioral despair (i.e., decreased forced swimming) (Wann et al., 2007). Our study declared that rats in the AMI group showed depression-like behavior, as performed by the reduced ability of movement in OFT and longer immobility time in FST. These findings implied that depression-like performance in rodents with MI was demonstrated by diverse behavioral tests.

Depression is recognized as a circuit disease influencing multiple encephalic regions connected in functional networks. The hippocampus, as a primary zone in the cerebral limbic system, has been identified as a major role in the pathological progress of depression. Many factors that may interact with hippocampal damage to trigger depressive episodes, neurotransmitter disturbance, and altered neurotrophic signaling are included (Kraus et al., 2017). The 5-HT hypothesis is supported by vast amounts of data that serotonin metabolism is altered in depression (Dell'Osso et al., 2016). The shunt of Try from 5-HT to Kyn formation is a dominating etiological factor of depression. Kyn was reported to be a proinflammatory metabolite in the neuroimmune signaling network mediating depressive-like behavior (Zhang et al., 2020). The Kyn/Try ratio, an indicator of the activation of the first step of the Kyn pathway, the elevation of which indicated a decrease in the conversion of tryptophan to 5-HT. Activation of the Kyn pathway *via* inflammation has been substantiated in clinical and preclinical research (Savitz, 2020; Troubat et al., 2021). Inflammation-driven alterations in kynurenine metabolic pathways result in substantial alterations in the metabolism of 5-HT. Our study showed a low content of 5-HT on day 7 postoperatively, accompanied by a rise in Kyn/Try ratio. Myocardial infarction might disturb tryptophan metabolism through the kynurenine pathway, thereby resulting in a decrease in 5-HT synthesis. PCF might change the expression of 5-HT directly *via* the kynurenine pathway, thus improving depression-like behaviors in AMI rats.

One of the most attractive features of the hippocampus is the unusual capacity for adult neurogenesis. In the sub-granular zone of the dentate gyrus (DG) of the hippocampus, newborn neurons are continuously generated, developed into mature neurons, and functionally integrated into the existing neural circuitry. It is now well established that adult hippocampal neurogenesis is decreased in rodent models of depression (Tanti et al., 2013). Proinflammatory cytokines are involved in immune system-to-brain communication by activating resident microglia in the brain. Activated microglia reduce neurogenesis by suppressing neuronal stem cell proliferation, promoting apoptosis of neuronal progenitor cells, and decreasing the survival of newly developing neurons and their integration into existing neuronal circuits (Cope and Gould, 2019). The process of neurogenesis is strongly stimulated by a brain-derived neurotrophic factor (BDNF), a neurotrophic factor that modulates functional and structural plasticity in the central nervous system, thus affecting dendritic spines and adult neurogenesis. A mass of studies reported the association of a decrease in BDNF mRNA and protein levels in the hippocampus with an increase in susceptibility to develop depressive disorders (Karege et al., 2002; Weinstock, 2017). For synaptic plasticity, we observed the morphology and number of neurons in the hippocampus through Nissl staining. The experimental results showed that the neuronal body of the hippocampus in the AMI group was lost. In our study, the effects of the AMI model on the expression of the synaptic-plasticity protein in the hippocampus were explored by western blotting. The decrease in BDNF might account partly for the depression-like behavior in AMI rats. The results were opposite for rats treated with PCF, although no significant statistical difference was found.

Some experimental studies, until now, have evaluated the anti-inflammatory efficacy of various drug therapies in depression after MI. Ge et al. demonstrated that Ginkgolide B significantly increased the 5-HT content in the brain median raphe nucleus and cortex *via* the reduction of IL-1β to ameliorate depression in MI mice (Ge et al., 2020). Wang et al. revealed that oral minocycline could prevent increases in plasma cytokines and microglia activation, thus causing some improvement in cardiac function and depression-like behavior (Wang et al., 2019). Our study focuses not only on the role of PCF in improving depression-like behavior after MI but also on the function of controlling the upstream switch of microglial inflammation. Microglia express pattern recognition receptors (PRR) that are designed to identify DAMP (e.g., S100A9) and mediate inflammatory responses. The administration of PCF led to a reduction of the S100A9 level, which meant that PCF might cut the pathological chain of the S100A9-microglial activation-inflammatory cascade from the early stage.

There are some laboratory achievements for the molecule compounds of components in PCF consistent with the inflammatory mechanism obtained in our study. The published literature showed that ferulic acid, an important active ingredient in *Chuanxiong Rhizoma*, was proven to be antidepressive *via* increasing monoamine neurotransmitter levels in the hippocampus (Zhang et al., 2011). Also, the antioxygenation property of ferulic acid was implicated in the alleviation of myocardial injury in ischemia-reperfusion rats (Liu et al., 2021). Our *in vitro* study found that ferulic acid had an inhibitory effect on S100A9-induced microglial inflammation, providing evidence for the mechanism underlining the anti-depressed function. Neocryptotanshinone, a natural product isolated from Salvia miltiorrhiza Bunge, showed anti-inflammatory effects by inhibiting NF-κB and iNOS signaling pathways in LPS-stimulated mouse macrophages (RAW264.7) cells (Wu et al., 2015). It is implied that a percentage of agents showed therapeutic effects similar to PCF in psycho-cardiology diseases. The future for active ingredients in PCF within standardized quality control ensures repeatable pharmacological action.

There are limitations to our study. Only a single group was set in this research instead of several dose groups. Although the efficacy of PCF in depression after AMI was performed and confirmed in preliminary experiments, the absence of multi-dose groups cannot ensure a dose-response relationship. S100A9 is a small calcium-binding protein of the S100 family that is expressed, in most biological settings, as a heterodimer complexed with its partner, S100A8. Future research concentrating on the functional and pathological difference between the monomer and heterodimer is urgently needed. Due to the limited sample in our experiments, no significant statistical difference was observed in the expression of some indicators between groups. Moreover, nothing but *in vitro* evidence was provided that S100A9 triggered microglial activation through the TLR4 pathway, yet animal experiments in which a biological metabolism was more similar to the human body remained absent.

## 5 Conclusion

Taken together, PCF, a modified TCM formula, promotes the recovery of cardiac function and improves depression-like behavior after MI. The possible mechanism involved in the protective effects of PCF *in vivo* includes the reduction of inflammation, apoptosis, and fibrosis in the myocardium, the inhibition of the Kyn pathway, and a boost of neurogenesis in the hippocampal tissue. Our results identify S100A9 as a promoter of macrophage/microglia inflammation, with a central role in depressive disorder induced by AMI. The concept of a common modifier driving both myocardial and hippocampal immune response to AMI is novel and of major significance for realizing the immunopathology of this disease. Indeed, the effects of short-term S100A9 blockade closely recapitulate the consequences of reduced inflammation on cardiac function and depression. PCF is also proved to be efficacious for targeting local and systemic inflammatory phases after MI. *In vitro* experiments conclude that protein S100A9 promotes the production of proinflammatory cytokines in microglia *via* TLR4, while PCF serum inhibiting the release of S100A9 may provide a therapeutic approach in microglial-mediated neuroinflammatory diseases. These findings provide scientific evidence for the cardioprotective and antidepressive effects of PCF, particularly in the process of suppressing macrophage/microglia inflammation.

## Data Availability

The datasets presented in this study can be found in online repositories. The names of the repository/repositories and accession number(s) can be found below: MetaboLights‐MTBLS4527. Supplementary data can be found online at http://proteomecentral.proteomexchange.org/cgi/GetDataset?ID=PXD027832. The mass spectrometry proteomics data have been deposited to the ProteomeXchange Consortium (http://proteomecentral.proteomexchange.org) *via* the iProX partner repository with the dataset identifier PXD027832.

## Glossary

AI,: apoptosis index
AMI,: acute myocardial infarction
Arg-1,: arginase-1
BDI,: beck depression inventory
BDNF,: brain-derived neurotrophic factor
BSA,: bovine serum albumin
BZ,: peri-infarct border zone
C34,: TLR4 inhibitor
CD68,: cluster of differentiation 68
DAMP,: damage-associated molecular pattern
DAPI,: 4,6-diamino-2-phenyl indole
DEPs,: differentially expressed proteins
DG,: dentate gyrus
DMEM,: Dulbecco-modified eagle’s medium
DMSO,: dimethyl sulfoxide
ECG,: electrocardiograph
ECL,: enhanced chemiluminescence
ELISA,: enzyme linked immunosorbent assay
FA,: ferulic acid
FBS,: fetal bovine serum
FST,: Forced swimming test
GABA,: γ-aminobutyric acid
GAD67,: glutamic acid decarboxylase-67
GAPDH,: glyceraldehyde-3-phosphate dehydrogenase
GC-MS,: gas chromatography-mass spectrometry
HE,: hematoxylin and eosin
HRP,: horseradish peroxidase
Iba1,: ionized calcium-binding adapter molecule 1
IL-1β,: interleukin-1β
iNOS,: inducible nitric oxide synthase
IOD,: integrated optical density
LAD,: left anterior descending
LC-MS/MS,: label-free liquid chromatography-tandem mass spectrometry
LVEF,: left ventricular ejection fractions
LVFS,: left ventricular fractional shortening
LViDd,: left ventricular end-diastolic inner diameter
LViDs,: left ventricular end-systolic inner diameter
LVEDV,: left ventricular end-diastolic volume
LVESV,: left ventricular end-systolic volume
Kyn,: kynurenine
NF-κB,: nuclear factor kappa-B
NO,: nitric oxide
OFT,: open field test
PCF,: psycho-cardiology formula (Shuangxinfang)
PEG400,: 40% polyethylene glycol 400
PRR,: pattern recognition receptors
PVDF,: polyvinylidene difluoride
RAGE,: receptor of advanced glycation end products
S100A9,: Ca2^+^ binding proteins belonging to the S100 family
SD,: standard deviation
SSRIs,: selective serotonin reuptake inhibitors
TCM,: traditional Chinese medicine
TLR4,: toll-like receptor 4
TNF-α,: tumor necrosis factor-α
Try,: tryptophan
TSPO,: translocator protein
TUNEL,: TdT-mediated dUTP Nick-End Labeling.
